# Care for technology dependent children and their relationship with the health care systems^1^

**DOI:** 10.1590/0104-1169.0258.2554

**Published:** 2015

**Authors:** Aline Cristiane Cavicchioli Okido, Márcia Maria Fontão Zago, Regina Aparecida Garcia de Lima

**Affiliations:** 2 PhD, RN, Escola de Enfermagem de Ribeirão Preto, Universidade de São Paulo, WHO Collaborating Centre for Nursing Research Development, Ribeirão Preto, SP, Brazil; 3 PhD, Associate Professor, Escola de Enfermagem de Ribeirão Preto, Universidade de São Paulo, WHO Collaborating Centre for Nursing Research Development, Ribeirão Preto, SP, Brazil; 4 PhD, Full Professor, Escola de Enfermagem de Ribeirão Preto, Universidade de São Paulo, WHO Collaborating Centre for Nursing Research Development, Ribeirão Preto, SP, Brazil

**Keywords:** Child, Family, Pediatric Nursing, Anthropology, Medical, Home Nursing

## Abstract

**OBJECTIVE:**

to understand the experience of care delivery to technology dependent children based on the mothers' experience.

**METHOD:**

exploratory study with qualitative approach, based on the theoretical framework of medical anthropology and the narrative method. Twelve mothers participated and, as the technique to obtain the narratives, open interviews were held at the participants' homes.

**RESULTS:**

the narratives were organized into three thematic categories: the family system, identifying the care forms, the association between popular and scientific knowledge and the participation of the social network; the professional system, which discusses the relations between professionals and family, the hegemony of the biomedical model and the role of nursing; and the popular system, presenting popular care practices like spirituality and religiosity.

**CONCLUSION:**

the study provided support for a health care project that takes into account the families' moral and symbolic values and beliefs in view of the illness of a technology-dependent child. The results found can contribute towards changes in the health work process, so that its foundation is guided not only by the biomedical model, allowing the integration of the sociocultural dimensions into the health care movement.

## Introduction

The term technology-dependent is used to describe children who need some device to compensate for the loss of some vital function and consequently to maintain their existence, such as a tracheotomy, mechanical ventilation and gastrostomy^(^ 1 ^)^. Technology-dependent children are a group with special health needs, characterized by clinical weakness, health care demands beyond those offered to children of the same age and, frequently, the need for technological devices^(^ 2 ^)^.

These children require a complex and continuous therapeutic and rehabilitation regimen, which implies the need to incorporate new knowledge and practices that are uncommon in the daily existence of their mothers, as the care goes beyond what is offered to a healthy child. These care practices are based on scientific knowledge^(^ 3 ^)^.

In that sense, "hospital care at home" is positive as they are extended, enhancing these children's quality of life. On the other hand, the families face an extreme emotional and social burden^(^ 4 ^)^. The maternal burden, the changes in the intra and extra-family dynamics, the social isolation and the financial impact are some of the challenges these families experience^(^ 5 ^)^.

Researchers have alerted to the need to reorganize the health system to improve health care for this emerging clientele, looking for support networks of professionals who deliver home and community-based care. They also indicate the importance of qualified and integrative care and the need to implement new support services to help these families to maintain care at home^(^ 2 ^,^ 6 ^)^. The institutions that deliver home care to this group of children should be accessible, deliver family and community-oriented care coordinated with other services and in a culturally effective manner^(^ 7 ^)^.

Departing from the premise that the family members are alone facing a completely new reality, including new knowledge and practices that need to be incorporated into the daily life, this paper was based on the theoretical framework of medical anthropology. This framework provides a theoretical structure to analyze the cultural factors that intervene in the health area, studying the way people explain the causes of the diseases, the types of treatment they believe in and use, thus permitting the recovery of the experience-based and symbolic dimension of the care experience for technology-dependent children^(^ 8 ^)^.

With regard to medical anthropology, the concept of Health Care System was highlighted, which affirms that the activities in search of health care are socially and culturally organized, composing a cultural system based on three intercommunicating sectors: family (or traditional), professional and folk. The family or traditional sector is the sphere in which the disease is defined, discovered and where the health care activities are started. The professional sector consists of scientific medicine and the folk sector comprises non-official cure professionals, such as faith healers, prayers and folk healers, among others^(^ 9 ^-^ 10 ^)^. A complementary context is the Explanatory Model of Disease, which is an articulated set of explanations about disease and treatment that permeate each Health Care System, including the professional explanatory model, which is scientifically based and refers to what the health professionals adopt and to the popular explanatory models, resting on the culture shared by all members of a given group^(^ 8 ^)^.

Thus, the theoretical choice is based on the possible interpretations of the ways of thinking and acting in view of the disease and care for the technology-dependent child, integrating the sociocultural aspects of the participating mothers. Therefore, based on the premises of medical anthropology, the challenge was raised to understand the explanatory models these mothers elaborate, which guide their perceptions and attitudes in view of the care experience for their technology-dependent children in a given health care system.

This study is justified by the lack of research focused on these clients and the need for their recognition in order to plan public policies and care strategies. The development of this study was motivated by the need to answer the following research question: what is it like to take care of a technology-dependent child? Based on these considerations, the objective of this article is to understand the experience of care delivery to technology-dependent children based on the mothers' experience.

## Method

An exploratory study with a qualitative approach was undertaken, based on the theoretical framework of medical anthropology and the narrative method. The participants were twelve mothers of technology-dependent children, intentionally selected based on a sample of 102 mothers^(^ 11 ^)^. The 102 mothers were identified by an active search in the city between October and December 2010, who participated in the first phase of the doctoral dissertation. The active search was undertaken in the primary health care network of a city in the interior of the State of São Paulo, at institutions that support children with special health needs and by the divulgation of the research in written and spoken communication media.

The number of participants was not determined *a priori, *but defined in the process of the obtaining the empirical data, until the data were considered sufficient to understand the study phenomenon. It was based on the following argument: when the selection of the participants in a qualitative research is intentional, the researcher's goal is to have a selected sample of participants who can contribute better to the research, selecting cases that are rich in information for the in-depth study^(^ 12 ^)^.

Hence, the intentional sample was aimed at constituting a heterogeneous sample, including mothers with distinct experiences, whether because of the socioeconomic conditions, marital situation or length of experience with the disease. Thus, the inclusion criteria were: being a mother and/or responsible for children up to 12 years of age who depends on some technological device to maintain their life and who live in the city of Ribeirão Preto. After the intentional selection, preliminary contact was made by telephone, inviting the selected mothers to participate in this research phase. None of them refused and the data were collected according to the participants' availability.

The empirical material was produced between May and October 2011. The narrative method was used, which was considered the most appropriate as it permits rescuing the knowledge, interpreting relevant experiences and obtaining the cultural meaning. The narratives are histories the participants tell, whose thoughts, emotions and experiences are data sources. The narrative privileges the narrators' subjectivity and represents their experiences^(^ 13 ^)^. The open interview was used as a technique to obtain the narratives. On the basis of these premises, the construction of the narratives started with the following question: "Tell me about your experience of taking care of your technology-dependent son/daughter."

The home environment was selected as the place of study, comprising 12 home visits with an approximate length of 90 minutes and performed by the same researcher. The researcher was a Ph.D. student with preliminary experience involving the research clientele. As the research involved human beings, the study was submitted to the Institutional Review Board, in compliance with National Health Council Resolution 196/96, and received approval under No. 405/2010. To preserve their anonymity, the mothers were identified according to the chronological order of interviews, as follows: mother 1, mother 2 and so forth.

The narratives were analyzed based on interpretive analysis. Thus, the concepts of medical anthropology guided the interpretation of the maternal experience in care for the technology-dependent children. It started with the organization of the empirical material, which contained the identification and fully transcribed narratives. Next, exhaustive reading took place, followed by the search for common words, phrases and behaviors, and possible differences, called "data coding". The intermediary analysis process consisted of the cross-sectional reading of the codes, grouping of the common and uncommon codes and identification of the empirical categories. Finally, the categories were regrouped and interrelated according to their similarities, thus constituting the thematic categories^(^ 14 ^)^.

As the health care actions are organized in a sociocultural manner and can be studied by means of the health care system. As this system is understood as a set of actions to cope with the disease, such as the search for its meaning, the way it is treated and the institutions involved in care^(^ 10 ^)^, the thematic categories present the meaning of the maternal experience in the care for the technology-dependent child, based on the health care systems: family system, professional system and folk system.

## Results and Discussion

To investigate the maternal experience in care for technology-dependent children, the participants' different social contexts were considered. The analysis was based on the complementation and enrichment each experience permitted. Therefore, some characteristics of the mothers of the technology-dependent children who participated in this study are summarized: mean age of 34 years; regarding education, seven mother had finished secondary education, two had finished primary education and three had not finished primary education; in terms of occupation, two mothers worked formally and the remainder were full-time caregivers, but five of them contributed to the family income by the sale of handicraft and food at their home; concerning the marital situation, eight mothers had a stable relationship and the remainder was divorced or single; as to the number of children, five mothers only had the technology-dependent child, whereas seven mothers had between three and seven children; the length of experience with child care ranged between one year and six months and 11 years. In relation to the technological devices that the children used, 11 children nedeed technological devices for eating, such as gastrostomy, jejunostomy or gastric probe; seven children used devices for breathing, such as tracheotomy, non-invasive mechanical ventilation and oxygen therapy; and three children used devices for elimination, including urinary relief catheter and intestinal washing. It is important to highlight that the 12 children used more than one technological devices concomitantly.

## The family system

It is in the family subsystem that the diseases are identified first and, after the biomedical diagnostic intervention and the establishment of treatment, the sick people return and, together with other members of the system, assess the appropriateness of the treatment and decide on what to do further. In this system, the mother is the main care provider and needs to gain skills and knowledge for this new care form. Nevertheless, the narratives reflect the difficulty to assimilate this new care, as it is hardly present in lay persons' daily life, demanding the incorporation of practices that are exclusive to the professional explanatory model: *At first, when he did the gastrostomy, I didn't even touch him. I was terrified of seeing that I couldn't assimilate that that was for him to eat, I looked at it and thought it caused pain, that it hurt, I was afraid.* (Mother 9)

A transaction starts between the lay and the professional explanatory model, considering a certain disease and the care required^(^ 8 ^)^. Thus, at each new meeting between mothers of technology-dependent children and care professionals, a new lay explanatory model is organized and gains a new meaning, constructing singular care for their children, permeated by biomedical notions related to complex health care, as well as by the cultural perceptions of the phenomenon experienced. The testimony of mother number four is in line with these assertions as the child was monitored by different health professionals, and the mother managed all care orientations and applied them according to her experience: *At first, we really got kind of lost and then I started creating my methods. I got completely lost, I got home totally distraught. Various professionals are involved, a neurologist, pediatrician, physiotherapist, speech [speech, language and hearing therapist], I lost my mind. But over time I created my own method, adapting the times.* (Mother 4)

In a study aimed at describing the inter-relation and communication in the health care sectors from the perspective of family caregivers of chronically ill patients, the results were similar, indicating that, although the professionals determine what care is to be delivered, the caregivers end up developing their own ways, corresponding to an association between folk and scientific knowledge. They concluded that the knowledge is interconnected and that the caregiver defines when to turn to one or to another care system^(^ 15 ^)^. In view of the willingness to provide what is best for the child, the care experience, initially considered as threatening, gains a new meaning over time and the practices are transformed, making the mothers calmer with regard to daily care management: *I did not have any experience when my child left the hospital. I didn't know how to take care, when I was going to aspire him I cried. There was nobody to help me, but I had no choice, either I learned or I lived at the hospital. Then we gave care, care, now, thanks God, we were able to control everything at home.* (Mother 12)

As regards the engagement of the other members of the family system, this is indirect care, in the form of emotional and financial support. According to the experience of mother number eight, the relatives do not feel prepared to take care of the child, in view of the complexity of the care that is required: *Look, I am travelling today and return tomorrow afternoon, do you stay? [referring to the family members]. Impossible. But they do help me, they support me, they always come here. They're here every week, everyone, everyone comes. They don't let me stay here alone, they're very concerned, everything. They're always sending gifts, whenever there's a party they call, they are very concerned with the family. They're just not prepared yet to take care of her.* (Mother 8)

In most of the mothers' experiences, however, a weakened and hardly participatory family social network was verified in the care process for the technology-dependent children, signaling the isolation of the nuclear family from the other members: *Nobody participates, I have no family, my mother doesn't live here, because we don't come from here, we come from Recife, so, from nobody, just from who lives here really. *(Mother 7)

When studying the experience of families living with cystic fibrosis, the researchers also pointed out that, in cases of chronic childhood illnesses, the mother becomes the main figure in the execution of care and management of therapy, which becomes a burden^(^ 16 ^)^. In that sense, the argument is supported that the women were socialized as caregivers and good mothers, so that care for the sick child represents a legacy of dedication, abnegation and moral obligation, which are closely related to cultural issues, values, beliefs, religion and worldview^(^ 3 ^)^.

As the family care system includes the sick individual, the family, the social network and the members of the community nearby^(^ 8 ^)^, one may say that the constitution of partnerships among women, mothers of children with special health needs, is also part of this system. Based on the narratives, an informal network of solidarity is observed among the mothers, who become close and help one another: *My family is like that, each person for himself and God for all. I don't get family support, that's something that strikes me. My family are the mothers, the people who help me.* (Mother 9)

The constitution of this partnership among the women, mothers of children with special health needs, can be understood by the legacy of solidarity that involves the female universe and empowers them for care^(^ 3 ^)^. The importance of the approximation of groups of people who live with the same situation is evidenced in studies involving mothers of children with cystic fibrosis^(^ 16 ^)^. These approximations permit emotional and instrumental support, sharing of experiences, information and material, as well as the completion of the gap created in the traditional family sector.

### The professional system

The family system is responsible for 70 to 90% of the health care activities and involves beliefs, practices, choices, decisions, roles and relationships^(^ 9 ^)^. Nevertheless, the severity of the clinical conditions and the complex demand for care these children require demands skill, linking the care practice with scientific knowledge, necessary for the prevention or minimization of problems, the identification of clinical alterations and agile intervention, if necessary. In that sense, it is observed that the mothers' narratives about the experience of the technology-dependent children's disease were elaborated according to the technical terms and in conformity with the professional explanatory models, confirming the hegemony of the medical sciences in the Western paradigm^(^ 17 ^)^.

This appropriation of the biomedical discourse is justified based on the following premise: during the interaction between professional and sick individual, the explanatory models are shared, but the professional class influences and molds, so that the patients and family members adjust themselves to the biomedical model of the disease. This influence can be related to the social power of the professional class, especially the medical class, due to its origin and training^(^ 8 ^)^.

The narrative of mother number 12 evidences the organization of her life around the professional system, that is, due to her son's convulsive crises that are hard to control. She and her family had moved three times already, always looking for a place that permitted better access to the health services: *We wanted to stay close to a service, we were very scared. Then they said it was good there, so we started looking for a house there, because they said the service Cuiabá was one of the best in Ribeirão.* (Mother 12)

The narratives outlined the therapeutic search trajectory, mainly in the professional system, but the narrated experiences showed that the care professionals often ignore the maternal knowledge gained in the course of their experience: *Because there are doctors who don't listen to you, they don't let the mother speak: There was a doctor who gave him unnecessary medication. His hand trembled, his fingers, it got continuous. I told the doctor she didn't have to medicate him, she ordered it and put him to sleep. She wouldn't listen. If you do the Valium [diazepam], you do it day and night because when he wakes up the trembles will come. *(Mother 12)

The relations between health professionals and patients should be constructed in a dialogical and non-hierarchical manner. Therefore, the community should be constructed from an intercultural perspective, that is, the health professionals should possess knowledge that differs from the lay culture; hence, the professionals do not need to give up their knowledge, but listen to the other. If there is no dialogue, when returning to the family care system, the patient will do whatever pleases him and what he considers most appropriate, depending on the way he interprets his disease^(^ 18 ^)^.

In that perspective, although the narratives were elaborated to recognize the need for health interventions, this disease situation often remits to an experience marked by discomfort and frustrations. Mother number 7 reported on the difficult acceptance of her husband to have the gastrostomy surgery: *When our daughter was about to put the probe, I almost got depressed, because he wouldn't accept it. I had to see a psychologist, if I had know I would have had it installed earlier, because I almost lost because she chocked that much. Her father wouldn't accept it and he was even worse than me, he even fought with the doctors.* (Mother 7)

In view of the above narrative, it is agreed upon that the medical practice should center on the individuals and their culture, so as to rescue the humanization of care and the integrality of health care. The health services should be "culturally sensitive", that is, the professionals need to be prepared to recognize the cultural differences among individuals in search of health care, thus permitting effective action in response to their problem, at risk of a partial approach of the problem and a lesser chance of being successful^(^ 19 ^)^.

Among the professionals in the professional sector, the narratives revealed that nursing, by means of the orientations provided in the course of the hospitalization and at discharge, was essential to construct these mothers' autonomy to deliver care to their children, facilitating the continuity of care at home: *When I got home, there's a nurse from whom I learned a lot. He taught me everything, he said: look, that's how you give medication, that's how you do it, that's how you aspire. He taught me everything.* (Mother 10)

Nursing plays a fundamental role in care for technology-dependent children and their family, assuming the commitment to support them in the transition process to the home and further monitoring^(^ 5 ^)^, but this training remains based on the biomedical paradigm, on the accomplishment of techniques and on the disease.

### The folk system

The popular system consists of non-professional cure specialists, strongly linked to the family system. As the professional system by itself is not capable of responding to the mothers' expectations and needs, they started to search for other care systems. In the following narratives, mother number four mentioned some treatments done in parallel to the traditional allopathic treatment, with a view to improving the child's neurological condition: *I am giving fish head. They taught me that it's good for the brain and I do it with that much faith that one day I'll get results, provided that I'm not getting them already. She continues: He's on a bioenergetic treatment now, applies clay to the head, I don't know if you're familiar with the curative power of clay. Jesus Christ used clay to cure the blind and, if you search on Google, the curative power of clay, as well as aesthetic, so, we are attempting to work here *[pointing at the son's head].* Put on clay, leave it for 2h. It makes a big mess.* (Mother 4)

Among other folk health practices, the mothers mentioned the use of teas and herbs to combat different problems: *You mash the mint, put in some water, strain it and give it to him. For the worms in the tummy. Make a very strong tea.* (Mother 9)

The practice of complementary and alternative therapies has gained strength in the current context, especially for children with chronic illnesses. These therapies can be defined as non-conventional approaches, adopted for therapeutic purposes, including phytotherapy, homeopathy, anthroposophic medicine and acupuncture, which can be used separately or associated with traditional medicine^(^ 20 ^)^.

Religion is another part of the folk system in the Health Care System. In this perspective, mother number five's narrative reveals this simultaneous participation during heart surgery, by means of vibrations and energies: *They help, send vibrations, energies and surgeries too. On the day of this surgery *[referring to the heart surgery]*, the people from the *[spiritist]* center met, everyone. I even remembered that they scheduled, at work, some had to leave their work, to be able to do the vibration at the time of the surgery.* (Mother 5)

The experiences of mothers number six and eleven also highlight the importance of religion in care for their technology-dependent children: *Then my mother's church, you know she's evangelical, went there, prayed for him. I couldn't even sleep. *(Mother 6) and *My son's in hospital, the entire church is praying. My son went through this, the minister comes immediately, wherever he is. The day he went to the ICU, it was a matter of minutes, the minister was there already. He was there. *(Mother 11)

Religion contributes to establish meanings for human existence. It is also a source of motivation, protection and a personal energy resource, helping to cope with the disease and death^(^ 21 ^)^. In that sense, mother number five adds: *The spiritist center made me very strong. Spiritualism helps you to accept in a comforting way. It was my strength, from where I got all of my strength. They never told me: we're going to cure your daughter, she'll walk, she'll talk. They don't promise cure, but they advise you on how it works, the reason for everything, my part and hers in this situation.* (Mother 5)

Spirituality and religiosity encourage the family, generating feelings of hope or acceptance of the condition the disease imposes. In a study on spiritual care in oncological pediatric nursing, the authors also concluded that religion and spirituality are sources of comfort and hope, helping to accept the disease of the child and adolescent^(^ 22 ^)^.

## Conclusion

In this study, we started from the premise that the mothers attribute meaning to the child's disease beyond the objective recognition and formal diagnosis. Hence, it was considered proper to support the analysis on these concepts, as they are more appropriate to the objectives, considering that this theoretical reference framework involves the understanding of the interfaces between folk knowledge and scientific medical knowledge about the disease and its causes, to be negotiated on in the cure process.

The study identified the need for further approximation among health professionals, children and their families. Hence, health professionals can better understand what it means to live with a chronic disease, how the disease becomes part of the daily life and how it influences their decisions. The intention in this study was to start a discussion on the theme, so as to mobilize the reflection of the members of the professional system and other subsystems involved in care about innovative strategies, guaranteeing high-quality home care for these children, as well as for the caregiving families.

It was also identified that the disease experience is mainly centered on the professional system, which nevertheless displays little problem-solving ability in care for the complexity involved in care for technology-dependent children, making the family and folk systems fundamental in the maternal experience. Hence, the interpretations can contribute towards changes in the health work process, so that it stops being exclusively based on the biomedical model and so that the sociocultural aspects are integrated in the health care movement. This reveals the need to value the spiritual support in the action planning of the professionals working with these clients. In that sense, the importance of nursing is highlighted to transmit this support, in view of its continuing presence in the situations where it is required.

Considering the results related to the importance of the nursing professionals and their generalist background, which considers not only individual care, but also collective and general care, the nurses are key persons to integrate these care systems. Therefore, this theme should be included on the list of study subjects as soon as the future nurses start their undergraduate programs.

The informal solidarity network of these mothers is highlighted, helping one another. In that perspective, formal actions are needed to allow these mothers to meet, with a view to strengthening the bonds of solidarity, as they share facilities and difficulties in view of the experience of having a child who requires care beyond what a healthy child demands. In addition, it is considered that this type of approximation grants visibility to sociocultural issues, which are hardly valued in the professional sector.

As regards the study limitations, the delimitation of a specific group of children with special health needs is appointed, i.e. technology-dependent children. This excerpt was necessary to optimize the time to accomplish the research. Nevertheless, it is known that, to contribute to the process of political changes that guarantee comprehensive care to these children and their families, an inclusion movement is needed. Another limitation refers to the dynamic nature of the narratives, which reported on the experiences these mothers are still going through, and which can therefore be reconstructed, based on the interaction and negotiation among individuals who share a certain sociocultural context.

Hence, this study supports reflections on how the professionals can develop personalized care, which takes into account the family members' cultural values and beliefs in view of the child's illness process. Further research is needed to support this construction.
